# Characterization of iRGD-Ligand Modified Arginine-Histidine-Rich Peptides for Nucleic Acid Therapeutics Delivery to αvβ3 Integrin-Expressing Cancer Cells

**DOI:** 10.3390/ph13100300

**Published:** 2020-10-10

**Authors:** Anna Egorova, Alexander Selutin, Marianna Maretina, Sergei Selkov, Vladislav Baranov, Anton Kiselev

**Affiliations:** 1 Department of Genomic Medicine, D.O. Ott Research Institute of Obstetrics, Gynecology and Reproductology, Mendeleevskaya Line 3, 199034 Saint-Petersburg, Russia; egorova_anna@yahoo.com (A.E.); marianna0204@gmail.com (M.M.); baranov@vb2475.spb.edu (V.B.); 2 Institute of Chemistry, Saint Petersburg State University, Universitetskii pr. 26, 198504 Peterhoff, Russia; 3 Department of Immunology and Intercellular Interactions, D.O. Ott Research Institute of Obstetrics, Gynecology and Reproductology, Mendeleevskaya Line 3, 199034 Saint-Petersburg, Russia; a_selutin@yahoo.com (A.S.); selkovsa@mail.ru (S.S.)

**Keywords:** nonviral gene delivery, siRNA delivery, receptor targeting, peptide-based carriers, integrin, iRGD

## Abstract

Efficient and specific delivery of nucleic acid (NA) therapeutics to tumor cells is extremely important for cancer gene therapy. Various therapeutic strategies include delivery of DNA-therapeutics such as immunostimulatory or suicide genes and delivery of siRNA-therapeutics able to silence expression of cancer-related genes. Peptides are a promising class of non-viral vehicles which are biodegradable and can efficiently condense, protect and specifically deliver NA to the cells. Here we designed arginine-histidine-rich peptide carriers consisting of an iRGD ligand to target αvβ3 integrins and studied them as vehicles for DNA and siRNA delivery to cancer cells. Combination of iRGD-modified and unmodified arginine–histidine-rich peptides during NA complexation resulted in carriers with different ligand contents. The NA-binding and protecting properties in vitro transfection efficiency and cytotoxicity of the DNA- and siRNA-polyplexes were studied and the most efficient carrier RGD1 was determined. The ability of the peptides to mediate specific intracellular uptake was confirmed inhuman cervical carcinoma (HeLa), human kidney (293T) and human pancreatic (PANC-1) cell lines with different αvβ3 integrins surface expression. By means of RGD1 carrier, efficient delivery of the herpes simplex virus (HSV-1) thymidine kinase gene to PANC-1 cells was demonstrated. Subsequent ganciclovir treatment led to a reduction of PANC-1 cells’ viability by up to 54%. Efficient RNAi-mediated down-regulation of GFP and VEGFA gene expression was achieved in MDA-MB-231-GFP+ breast cancer and EA.hy926 endothelial cells, respectively, by means of RGD1/siRNA polyplexes. Here we demonstrated that the peptide carrier RGD1 can be considered as promising candidate for development of NA therapeutics delivery systems useful in cancer gene therapy.

## 1. Introduction

Gene therapy is an attractive approach to treat diseases by compensating for gene defects or regulating gene expression by silencing unwanted genes. The significant research area in gene therapy is related to the development of strategies for cancer treatment [1]. Currently, the mortality rate from cancer disease is significantly high. In addition, cytostatics, currently used in anticancer therapy, induce the appearance of new chemotherapeutically resistant tumors in previously treated patients [2]. An alternative approach—cancer gene therapy uses various therapeutic strategies that include immunostimulatory gene and vaccine delivery, suicide gene therapy (DNA delivery), anti-angiogenic therapy aimed at silencing of pro-angiogenic gene expression (siRNA delivery) [3,4]. 

One of the main problems for gene therapy implementation remains the lack of effective and safe ways for targeted gene delivery to the cells and tissues [5]. Nucleic acid (NA) delivery vehicles have to meet the following requirements: to condense and protect NA efficiently, specifically enter the cells, prevent NA degradation and also be non-toxic and biodegradable [6,7]. Viral vectors are the most efficient gene delivery vehicles and play an important role in gene therapy [8]. However, the relatively small capacity for therapeutic NAs, the difficulty in receiving good quality formulations and safety concerns about viruses prompt the development of alternative systems to viral vectors [1].

Peptides are popular carriers for non-viral NA delivery in cancer therapy approaches and offer the potential for gene delivery in an efficient, safe and specific manner [9]. In fact, multicomponent peptide-based carriers can be suggested as “artificial viruses”—non-viral vectors that mimic viruses but lack their limitations. The possibility to modify the carrier structure and amino acid composition allows the inclusion of functional groups to overcome the extra- and intracellular barriers [6]. Different peptide modules can be designed to obtain multifunctionality of the carriers. The most promising arginine-rich peptide modules can not only neutralize the negative charge of NA, but possess a cell membrane translocation ability associated with guanidine groups which can interact with surface glycosaminoglycans [10]. Endosomal entrapment, being one of the most formidable barriers of NA delivery, can be overcome by modification of peptide-based vectors with histidine residues which helps the NA release from endosomes by their buffering with a pKa of 6.0 [11]. The gene delivery success could also be accompanied by severe side effects due to the therapeutic cargo accessing other healthy tissues [12]. Thus, efforts need to be focused on the development of targeted NA delivery systems for accumulation specifically at the tumor site. During tumor growth and metastasis, expression of various integrins is highly increased in the tumor vasculature and certain tumor cells, but is at much lower or undetectable levels on normal resting vessels. An in vivo phage display analysis has identified peptides that are selectively located on tumor blood vessels. As a result, Arg-Gly-Asp (RGD) peptide motif was detected, selectively binding to αvβ3 integrins. Furthermore, peptides and antibody antagonists of αvβ3 integrins could block tumor angiogenesis and tumor growth without affecting normal tissue [13]. Integrins of αvβ3 are directly involved in the formation of lung, mammary and prostate gland cancer, pancreas cancer, as well as participating in the process of tumor metastasis [13,14]. These make them promising targets for anticancer gene delivery. 

Previously, peptide ligands containing the RGD sequence have been developed for the treatment of various tumors [15]. The RGD peptides’ conformation strongly influence the RGD–integrin recognition. Cyclic RGD peptides demonstrate higher specificity for integrins and binding affinity in comparison with linear ones [16]. To date, several types of NA delivery vehicles have been extensively studied, including RGD ligand-modified cationic polymers, cationic liposomes and peptide-based vectors. Integrin-targeted cationic polymers possess endosome buffering and NA loading capacity, but their cytotoxicity is too high and their biocompatibility and biodegradability need to be improved. Cationic liposomal and peptide-based vectors have been developed to overcome these disadvantages, because they have better biocompatibility and can be easily degraded [17]. Previously, a number of authors developed cyclic RGD-modified cationic peptides. The peptides bound to NAs were effective for in vitro studies; moreover, an in vivo distribution assay showed preferred tumor localization of the complexes [18]. Recently, a novel RGD-containing peptide has been proposed as targeting moiety with tumor penetrating ability [15]. This ligand is a peptide iRGD (CRGDK/RGPD/EC), cyclized on cysteine residues. After interacting with the tumor vascular endothelium integrins, iRGD undergoes proteolytic cleavage followed by the formation at C-terminus of a truncated CRGDK/R peptide that binds to neuropilin-1 and the activates transport pathway inside the tumor stroma [19]. Such a dual sequential binding system provides high specificity and efficiency for the tumor delivery of therapeutic NAs. Application of iRGD as targeting moiety is perspective for peptide-based non-viral vectors; however, to date, a few iRGD-modified peptides have been studied as vehicles for NA therapeutics delivery. Feasibility of this approach was demonstrated recently in RNAi treatment of a pancreatic cancer model and breast cancer cells [20,21].

In present study we demonstrated versatility of iRGD-modified peptide vectors in therapeutic pDNA and siRNA delivery in vitro. An arginine–histidine-rich peptide consisting of an iRGD ligand (R_9_H_4_CRGDRGPDC) was designed and studied as vehicle for NA therapeutics delivery to cancer cells. Before the experiments, the formation of an intramolecular disulfide bond was carried out to obtain a carrier modified with the cyclic ligand. Combining of iRGD-modified and unmodified (R_9_H_4_) peptides during NA complexation resulted in two more carriers with different ligand contents: 50 mol% and 10 mol% of iRGD-modified peptide. The physico-chemical properties, transfection efficiency and cytotoxicity were investigated in details. The ability of the peptides to mediate specific intracellular uptake was evaluated in cell lines with different amounts of αvβ3 integrins on the cell surface. Moreover, efficiency and specificity of targeted NA delivery was demonstrated. Therapeutic potential of the developed carriers was demonstrated in suicide gene therapy experiments on cancer cells and in RNAi-mediated down-regulation of VEGFA gene expression in endothelial cells. 

## 2. Results and Discussion

### 2.1. Design of the Carriers

In the present study, DNA- and siRNA carriers were developed by cyclization of a linear iRGD ligand sequence conjugated with arginine-rich peptide. The RGD1 carrier comprises the ligand motif RGDRGPDC (iRGD) interacting with integrins αvβ3 on the cancer cell surface and then with neuropilin-1 after its truncation, thereby reaching the deepest tumor layers [19]. The location of the iRGD motif meets the requirement of C-terminal exposure for neuropilin-1 binding and the motif is separated from the DNA-binding sequence R9 by four histidine residues needed for NA release from endosome [19]. Weakly charged histidine residues also serve as a spacer between theiRGD ligand and NA-binding part of the carriers. The earlier studies of peptide ligands showed that the effectiveness of cell binding directly depends on the distance between the ligand moiety and another part of the carrier [6]. The cyclic iRGD ligand was obtained by the formation of intramolecular disulfide bonds. An RGD0 carrier (R_9_H_4_) was used as a ligand-free control. The RGD2 carrier was obtained by combining 50 mol% of RGD1 peptide and 50 mol% of RGD0 peptide, whereas the RGD3 carrier was produced by combining 10 mol% and 90 mol% of ligand-modified and control peptides, respectively. Designed carriers are illustrated in Table 1.

### 2.2. Evaluation of NA-Binding and NA-Protective Properties of the Carriers

**Figure 1:**
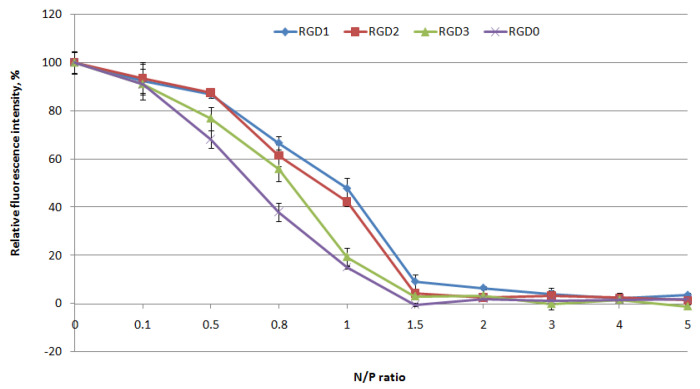
DNA binding studies by ethidium bromide (EtBr) exclusion from complexes of DNA and Arg-Gly-Asp (RGD)1, RGD2, RGD3 and RGD0 carriers. Values are the mean ± SD of the mean of triplicate experiments.

DNA binding by the peptide-based carriers was studied using ethidium bromide exclusion assay at different peptide NH3+/DNA PO4- (N/P) ratios (0.1–5) of the peptides to a plasmid DNA. Peptide binding to DNA leads to prevention of ethidium bromide intercalation and a decrease in fluorescence as seen at N/P ratio 1.5/1 for the vehicles (Figure 1). These results demonstrate that peptide modification with an uncharged ligand does not influence DNA binding ability. 

**Figure 2:**
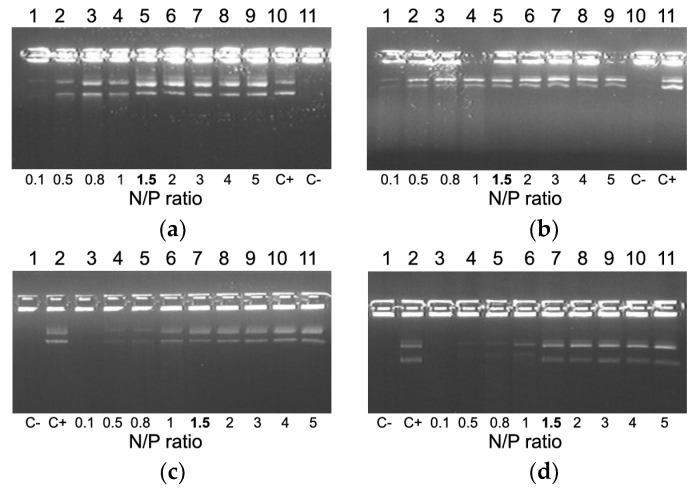
DNase I protective ability of DNA-complexes formed with RGD1 (**a**), RGD2 (**b**), RGD3 (**c**) and RGD0 (**d**) carriers. NH3+/DNA PO4- (N/P) ratio in bold indicates the beginning of DNA protection. C−, “naked” plasmid DNA treated with DNase I, C+, untreated plasmid DNA.

DNA integrity was estimated by means of a DNase I protection assay. It can be seen in Figure 2 that naked DNA is fully degraded after incubation with DNase I. Moreover, we found that an increase in charge ratio of DNA/peptide complexes resulted in better DNA protection. All the carriers exhibit full DNA-protection starting from N/P ratio 1.5/1 (Figure 2). 

**Figure 3:**
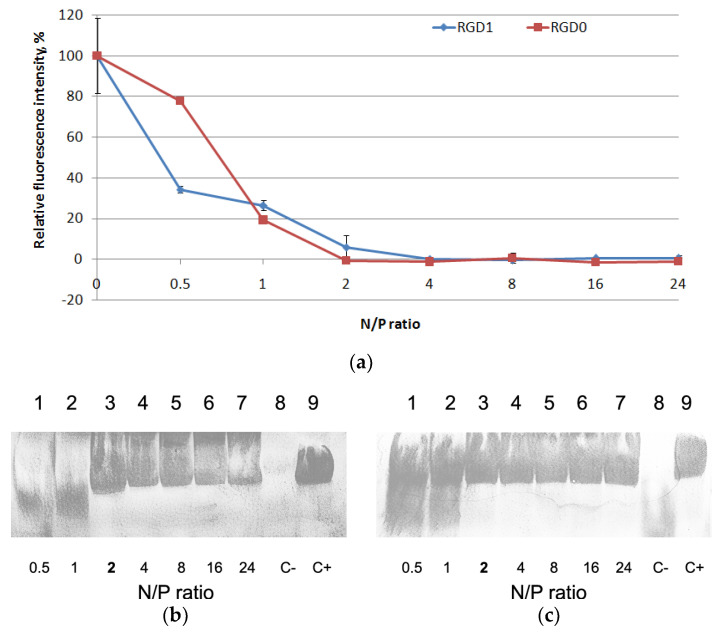
SybrGreen exclusion from complexes of siRNA and RGD1 and RGD0 carriers (**a**). A total of 100% of fluorescence intensity corresponds to free siRNA stained with SybrGreen. The data are shown as the mean ± SD RNase A protective ability of siRNA-complexes formed with RGD1 (**b**) and RGD0 (**c**) peptides. The polyplexes containing 0.2 μg of siRNA were treated with 100 ng of RNase A for 30 min at 37 °C. C− is siRNA treated with RNase A; C+ is untreated siRNA. N/P ratio number in **bold** indicates full defense from RNase A.

RNAi targeting of cancer-related transcripts is one of the most potent approaches in cancer gene therapy and the RGD1 carrier (as fully modified with an iRGD moiety) has been chosen to test its properties as siRNA delivery system [22]. It is known that complexes with siRNA can significantly differ from DNA-complexes, so protocols for siRNA delivery have to be developed separately [6]. The carriers were studied for siRNA binding using aSybrGreen exclusion assay at the various N/P ratios ranging from 0.5 to 24 of the peptides to siRNA. Peptide binding to siRNA led to SybrGreen exclusion and to decrease in fluorescence as seen at N/P ratio 2/1 for both carriers (Figure 3a). Thus, similarly to the case with DNA-polyplexes, the modification of the peptides with an iRGD ligand did not influence siRNA binding properties. 

We assessed the siRNA integrity using an RNase A protection assay (Figure 3b,c). After the treatment of naked siRNA by RNase A, it was not detected due to degradation. An increase in N/P ratio led to better siRNA integrity. The results showed that RGD1 and RGD0 carriers can provide siRNA with effective protection against RNase degradation. No difference in RNase A resistance was registered and all the carriers were able to protect siRNA at N/P ratio 2/1 (Figure 3b,c). These results are consistent with the data obtained in siRNA-binding studies (Figure 3a). 

Thus, we could suggest that inclusion of the uncharged iRGD ligand into the carrier’s composition does not influence the NA protection and does not interfere in the electrostatic interaction between the oligoarginine and NA. In addition, non-participation of the iRGD moiety in the NA-binding and protection can likely lead to successful interaction of the ligand with αvβ3 integrins on the cell surface and, therefore, to receptor-mediated uptake of the polyplexes.

### 2.3. Relaxation of NA/Peptide Complexes by Polyanions

**Figure 4:**
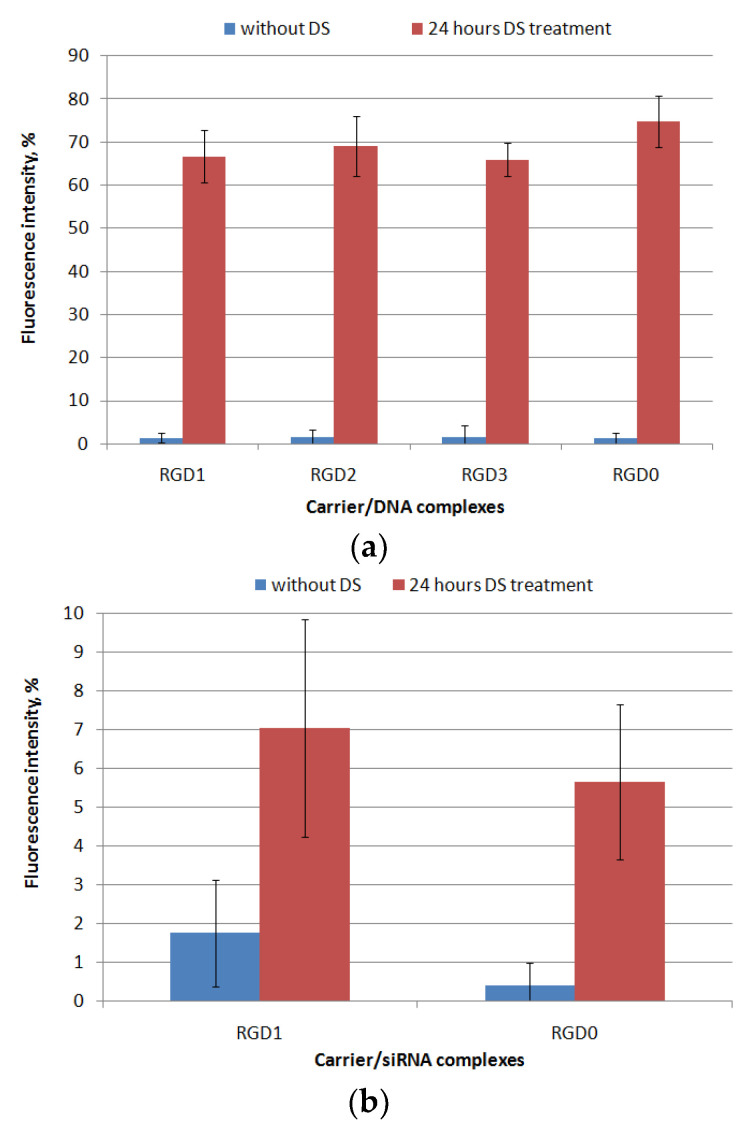
Relaxation of peptide/DNA (**a**) and peptide/siRNA complexes (**b**) at N/P ratio 8/1 after 24 h of DS treatment (three-fold charge excess). A total of 100% of fluorescence intensity corresponds to free DNA stained with EtBr or free siRNA stained with SybrGreen. Values are the mean ± SD of the mean of triplicate experiments.

It is known that extra- and intarcellular polyanions, e.g., glycosaminoglycans (GAGs) and RNA are disruptive for NA/peptide complexes [6]. These interactions can affect the transfection efficiency because of DNA- and RNA-complexes’ disassembly outside the cells whereby its intracellular internalization could be difficult. However, the high density of carrier/NA polyplexes may prevent NA release inside the cells, which also reduces the transfection efficiency. It was previously shown that RNA molecules, as well as proteins in the cytoplasm, contribute to the disassembly of NA-complexes inside the cell [6]. In order to evaluate the resistance of the polyplexes to the components of the extracellular matrix, we incubated the DNA- and siRNA-polyplexes with dextrane sulfate (DS) for 24 h in three-fold charge excess. The results are demonstrated in Figure 4. After 24 h of incubation with DS, DNA-polyplexes formed with RGD1, RGD2, RGD3 and RGD0 peptides were relaxed up to 65–75% of free DNA-dye fluorescence intensity. Thus, these RGDs/DNA polyplexes were susceptible to interaction with extracellular glycosaminoglycans, but the complexes’ disassembly was not complete. Thus, a necessary balance between the degree of NA release and the density of the formed complexes was maintained, which is important because controlling of complexation/decomplexation of polycation-based DNA-polyplexes is known to be a key factor in effective transfection along with their size and zeta-potential [23]. It is important to notice that the uncharged ligand did not contribute to degree of the complexes’ relaxation by polyanions as was expected. 

Similarly, resistance to polyanions was evaluated forRGD1 and RGD0 siRNA-polyplexes. We found that after the incubation the siRNA-polyplexes were only slightly relaxed (Figure 4b). It can be supposed that slow disassembly of the complexes may result from tight siRNA packing by arginine-rich peptides. In addition, interactions between GAGs and polyplexes are dependent on the GAG’s type [24]. Previously, we have already described that siRNA-complexes formed with oligoarginine carriers can be insensitive to the relaxation by DS [25]. At the same time, an addition of the uncharged RGD ligand to carrier did not reduce the charge density, and, as a result, did not weaken their siRNA-packing abilities. 

### 2.4. Measurement of Size and Zeta-Potential of Peptide/NA Complexes

**Figure 5:**
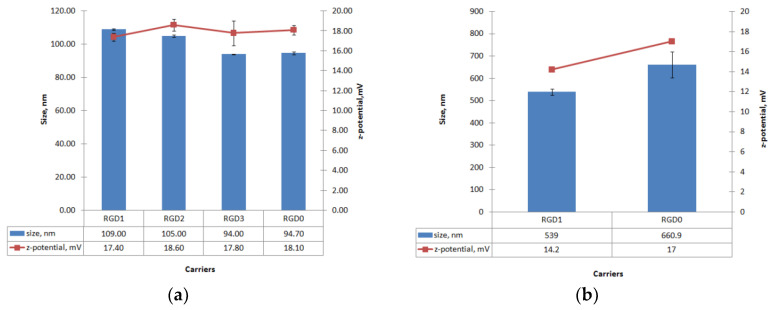
Size and zeta-potential of carrier/DNA (**a**) and carrier/siRNA (**b**) complexes at N/P ratio 8/1. The data are shown as the mean ± SD

Size and zeta-potential values were measured for RGD1/DNA, RGD2/DNA, RGD3/DNA and RGD0/DNA complexes and RGD1/siRNA, RGD0/siRNA complexes at an 8/1 charge ratio (Figure 5). The DNA-polyplexes’ sizes ranged between 90 and 110 nm. The size of DNA-polyplexes showed a tendency to increase as ligand content in the carrier increased, which might be a result of the ligand decoration of the polyplexes. However, the differences between ligand-modified and unmodified DNA-complexes were only marginally significant (0.03 < *p* < 0.05). Moreover, the in case of the RGD3 vehicle, it can be seen that its ligand part does not influence the size of RGD3/DNA complexes. It is important to notice, that the resultant sizes of the polyplexes allow their internalization by αv-integrin-mediated clathrin-dependent and independent endocytic pathways [13,26]. 

The zeta potential of the DNA-polyplexes ranged between +17 and +19 mV. No significant differences in zeta-potential between DNA complexes with ligand-modified RGD1, RGD2, RGD3 and unmodified RGD0 carriers were found. According to these data, the iRGD ligand does not appear to contribute to the zeta-potential of peptide/DNA complexes and, similarly, does not influence the DNA-binding and DNA-protective properties (Figure 1, Figure 2 and Figure 3). It is known that highly positive zeta-potential of NA-polyplexes promotes cellular uptake due to better binding of negatively charged plasmalemma with subsequent unspecific endocytosis [6]. However, the relatively low zeta-potential of the resulted polyplexes along with their good cell penetration ability allows us to suggest that the ligand-modified complexes might penetrate into αvβ3-positive cells not only through electrostatic interactions with plasmalemma but primarily by means of αvβ3-integrin-mediated endocytosis (Figure 5). 

Similarly, particle size and zeta-potential values were measured for the siRNA-polyplexes formed at N/P ratio 8/1 (Figure 5b). The particle size of the siRNA-polyplexes was found ranging from approximately 500 to 700 nm and had a tendency to decrease when ligand content was included in the carrier. This result indicates that the inclusion of the uncharged ligand part in the carrier composition slightly decreases siRNA-polyplexes’ size. On the other hand, siRNA-polyplexes, as expected, had bigger sizes than DNA-polyplexes. The differences in the sizes of DNA- and siRNA-complexes may be due to their significant structural differences. The interaction of DNA with polycations, as is known, leads to its condensation [27]. The possibility of condensation mainly depends on size as well as on persistent length of the molecule. The RNA molecules less than 260 bp behave like a “rigid rod” and cannot be condensed [6]. Thus, 21-nucleotide siRNA interaction with polycations can lead to incomplete encapsulation and formation of bigger complexes. In addition, the polycations were shown to have a much lower stoichiometry of binding to oligonucleotides than to DNA [6]. Importantly, the size of polyplexes may determine their internalization pathway. The clathrin-mediated endocytosis is typical for complexes with size lower than 200 nm, whereas the particles of 500 nm in size and upper enter cells predominantly by caveolae-mediated internalization [26].

The zeta potentials of the siRNA-polyplexes ranged between +14 and +17 mV, with minimal values for ligand-modified RGD1/siRNA and maximal for control RGD0/siRNA complexes. This suggests that the uncharged ligand content could somehow decrease surface charge of the polyplexes. However, despite slightly different size and Z-potential values, both ligand-modified and ligand-free carriers showed equal siRNA-binding and siRNA-protective efficiency (Figure 2 and Figure 3). Thus, the results of this experiment contribute to an understanding of the properties and structure of siRNA-complexes formed with unmodified and uncharged ligand-modified carriers.

### 2.5. Cytotoxicity Evaluation of Peptide/NA Complexes

The presence of αvβ3 integrins was detected on the surface of several cell lines by flow cytometry analysis. Cells were assayed using monoclonal antibodies to CD51/CD61. As shown, 293T cells expressed almost no αvβ3-positive cells (3% ± 0.5%), whereas the HeLa cell line expressed a considerably higher amount of αvβ3 integrins than the 293T cells and the percentage of CD51/CD61-positive HeLa cells was about 18.5% ± 2% (Figure S1). The PANC-1 cell line overexpressed these integrins and 34.5% ± 3% of the cells were CD51/CD61-positive. All the obtained data are consistent with previous studies of other researchers [28,29].

**Figure 6:**
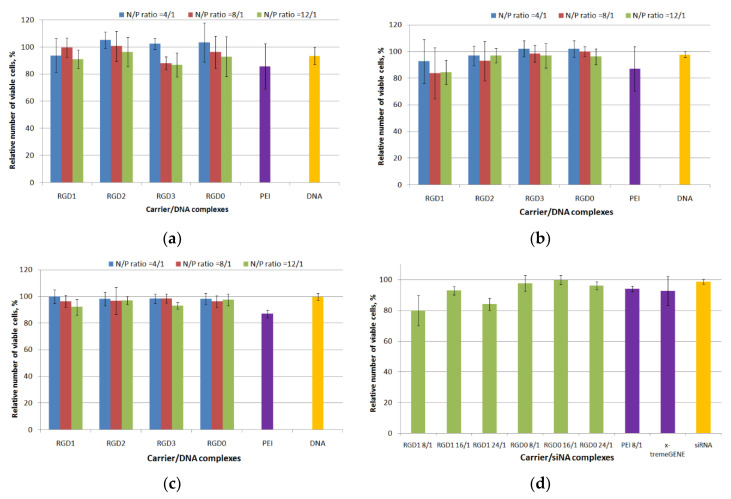
Cytotoxicity evaluation of carrier/DNA complexes in human pancreatic (PANC)-1 (**a**), human cervical carcinoma (HeLa) (**b**) and human kidney (293T) (**c**) cells and carrier/siRNA complexes in MDA-MB-231 (**d**) cells by the Alamar blue assay. Values are the mean ± SD of the mean of triplicateexperiments.

The absence of toxic effects from the carrier/NA complexes on cells is one of the important characteristics of an efficient carrier [30]. Several mechanisms are known by which polyplexes could exhibit cell toxic effects: destabilization of the extra- and intracellular membranes, interference with cellular processes through the interaction with proteins and RNAs [6]. Cytotoxicity of the DNA polyplexes at three charge ratios (4/1, 8/1 and 12/1) was determined in αvβ3-positive PANC-1 and HeLa cells using the Alamar blue assay. Also cytotoxicity of free carriers was evaluated in the same cell lines (Figure S2). In the PANC cell line peptide/DNA complexes showed low cell toxicity, that was similar to that of free DNA (Figure 6a). At a 12/1 charge ratio polyplexes became more toxic; however, their cytotoxicity was not higher than that of PEI/DNA complexes. In HeLa cells the percentage of viable cells after incubating with RGD2/DNA, RGD3/DNA and RGD0/DNA polyplexes was comparable to that of free DNA and intact cells. RGD1/DNA complexes at N/P ratios 8/1 and 12/1 were more toxic compared to RGD2/DNA, RGD3/DNA and RGD0/DNA polyplexes, however no significant difference was found between cytotoxicity of the studied polyplexes (Figure 6b). It should be noted that in 293T cells we did not observe cytotoxicity in all variants of DNA-polyplexes with the exception of PEI/DNA complexes (Figure 6c). The slight toxicity of RGD-polyplexes might be due to effect of the ligand moiety in the carrier’s composition. In early studies of the αvβ3 integrins’ ligands their possible toxic effects were demonstrated [31]. The RGD ligand binding to αvβ3 integrins—the cell adhesion receptors—could lead to cells detaching from the substrate, thereby resulting in disturbance of the cell cycle and metabolism processes and may lead to cell death. Nevertheless, the cytotoxicity of RGD1/DNA complexes did not exceed 20% and was not higher than that of PEI/DNA polyplexes (Figure 6b). It is important to notice that a general association between cytotoxicity and transfection efficiency suggests that a degree of membrane-damage is inevitable when effective carriers mediate DNA access to the cytoplasm. Successful transfection can rely on the correct balance between gaining the required access of DNA into the cytoplasm and causing excessive cell damage [32,33].

Cell viability after incubation with carrier/mock siRNA and PEI/mock siRNA polyplexes at different charge ratios (ranging from 8/1 to 24/1) was studied in MDA-MB-231 cells (Figure 6d). It was found that siRNA-polyplexes exhibit no cytotoxicity with the exception of RGD1/siRNA complexes formed at 8/1 and 24/1 that were slightly more toxic than naked siRNA (*p* < 0.05). At the same time, the amount of living cells after treatment with the complexes was not lower than 80%. These results showed that the studied polyplexes did not inflict significant cellular damage as well as their respective DNA-complexes (Figure 6a–c).

### 2.6. Cellular Uptake of Peptide/DNA Polyplexes 

**Figure 7:**
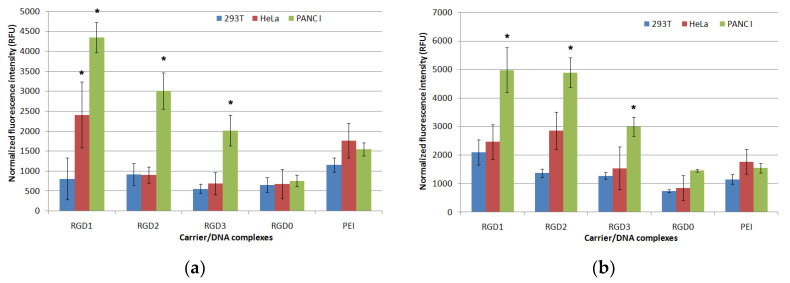
Normalized fluorescence intensity of PANC-1, HeLa and 293T cells after uptake of RGD1/DNA, RGD2/DNA, RGD3/DNA and RGD0/DNA polyplexes at 8/1 (**a**) and 12/1 (**b**) charge ratios labeled with YOYO-1. * *p* < 0.05 compared to RGD0/DNA complexes.

In order to evaluate cellular uptake of peptide/DNA complexes, we formed YOYO-1 labeled polyplexes at 4/1, 8/1 and 12/1 charge ratios. Uptake was assessed on HeLa, 293T and PANC-1 cells by flow cytometry. Living cells were analyzed. Normalized fluorescence intensity was measured in each sample (Figure 7). At a 4/1 charge ratio no significant difference in the uptake of ligand-modified and non-modified DNA polyplexes by PANC-1, HeLa and 293T cells was found (data not shown). In PANC-1 cells RGD1/DNA complexes at N/P ratios 1/8 and 1/12 had the highest penetrating ability. A decrease in the carrier penetration efficiency correlated with a decrease in the amount of the ligand part in the complexes. Thus, RGD1/DNA polyplexes penetrated 1.5 and 1.1 times more efficiently than RGD2/DNA complexes and 2.2 and 1.6 times more efficiently compared to RGD3/DNA polyplexes formed at 8/1 and 12/1 charge ratios, respectively. Complexes of DNA with RGD0 peptide were less effective in comparison with ligand-containing polyplexes and also with PEI/DNA complexes. The decreased uptake rate of RGD0 polyplexes might be due to the absence of cellular receptor binding ability, thereby preventing their endocytosis. Thus, the results obtained showed that the cell penetration efficiency directly depends on the amount of ligand in the polyplexes. In HeLa cells the penetration efficiency was lower. Only polyplexes formed with DNA and RGD1 at the 8/1 and 12/1 N/P ratios and with DNA and RGD2 at a 12/1 ratio were takenup by the cells more efficiently than the RGD0/DNA complexes. The data obtained are consistent with previous results indicating that a presence of the αvβ3 integrin ligand in the carrier composition significantly increases cellular uptake of NA-complexes [34]. For 293T cells, no differences in penetration efficiency of ligand-modified and unmodified polyplexes were found. Thus, we observed a direct and statistically significant correlation between an efficiency of cell penetration by the complexes and the amount of αvβ3 integrins on the cell surface. These results highlight the ability of RGD-modified carriers to provide targeted NA delivery. Therefore, the iRGD-containing DNA complexes could be takenup by αvβ3-positive cancer cells with limited side effects in normal cells.

### 2.7. In Vitro Transfection Studies of DNA-Polyplexes

**Figure 8:**
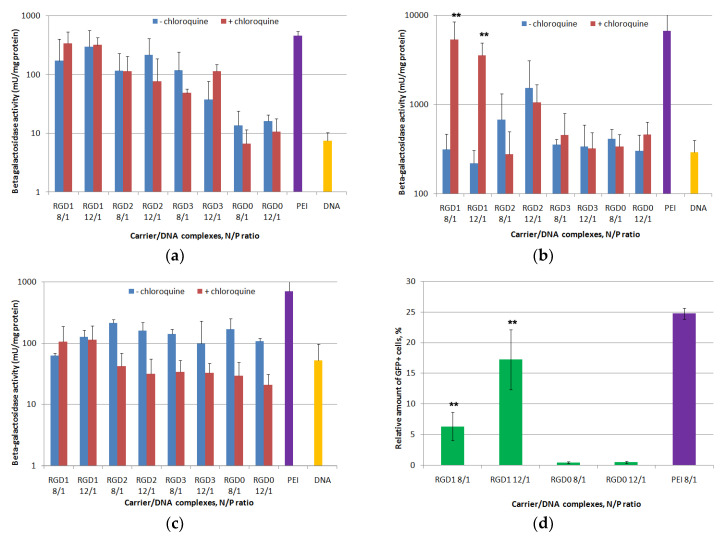

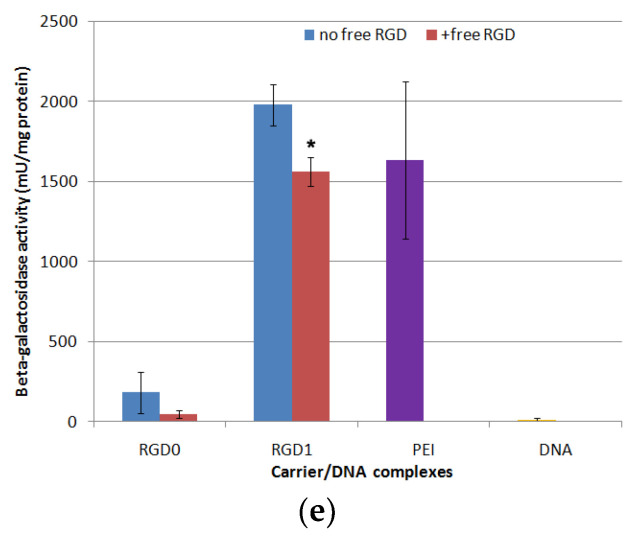
DNA transfection efficacy evaluation. The PANC-1 (**a**), HeLa (**b**) and 293T (**c**) cell lines were transfected with RGD1/DNA, RGD2/DNA, RGD3/DNA and RGD0/DNA polyplexes at charge ratios of 8/1 and 12/1, pCMV-lacZ plasmid alone and PEI/DNA. The transfection experiments were performed in the absence or presence of chloroquine. The PANC-1 cell line (**d**) was transfected with complexes of pEXPR-IBA5-eGFP plasmid and RGD1 and RGD0 carriers with charge ratios of 8/1 and 12/1. PEI polyplexes at an 8/1 charge ratio wereapplied as the control. Competitive transfection (**e**): the PANC-1 cells were transfected with RGD1/DNA and RGD0/DNA complexes at charge ratios of 8/1, pCMVlacZ plasmid alone and PEI/DNA. A free cyclo(RGDfK) ligand was added to the cells to compete with the complexes for binding of αvβ3 integrins. Reporter lacZ gene expression is given as milliunits (mU) per milligram of protein. GFP gene expression is given as a percentage of GFP-positive cells according to cytometry data. Values are the mean ± SD of the mean of triplicate experiments. * *p* < 0.05, ** *p* < 0.01 compared to RGD0/DNA complexes or compared to untreated complexes.

PANC-1, HeLa and 293T cells were used for in vitro transfection. The N/P ratios 8 and 12 were tried based on data from cellular uptake studies (Figure 8). In order to evaluate peptide endosomolitic properties, the transfection experiments were carried out in the absence and in the presence of endosomolytic agent chloroquine which is known to destabilize membranes during the transfection process and to prevent endosomal lysis of complexes [35]. Without chloroquine, transfection efficacy in PANC-1 cells of ligand-modified DNA complexes with RGD1 and RGD2 in all the studied charge ratios and RGD3 at the 8/1 N/P ratio was 10–20-fold higher compared to unmodified RGD0/DNA polyplexes (Figure 8a). Moreover, in some cases transfection efficacy of the RGD1 carrier was comparable to that of PEI. Similar findings were observed in PANC-1 cells for RGD1-polyplexes bearing plasmid with the GFP gene. The polyplexes at the 8/1 charge ratio provided 6.3% of GFP-positive cells, whereas at the 12/1 charge ratio the amount of GFP-positive cells was 17.2% (Figure 8d). According to GFP gene transfection results, efficacy of RGD1/DNA polyplexes was 16-37-fold higher compared to control RGD0/DNA complexes. This fact strongly supports the specificity of RGD carriers for the αvβ3 integrins. However, the β-galactosidase activity after cell transfection by RGD3-polyplexes formed at the 12/1 charge ratio was lower and did not differ from that of RGD0-polyplexes (Figure 8a). Chloroquine addition to transfection media did not lead to increase intransgene activity mediated by DNA complexes formed with RGD1, RGD2 and RGD0. However, thepresence of chloroquine in transfection experiments with RGD3/DNA polyplexes at the 12/1 charge ratio led to a significant increase in efficiency that was also higher in comparison with control RGD0/DNA complexes (Figure 8a). Inefficient endosomal escape is one of the most probable factors that contributed to the poor transfection by the polyplexes [32]. However, the results obtained in PANC-1 cells support the specificity of gene delivery mediated by ligand-modified carriers.

Further evaluation of the transfection specificity was carried out with HeLa cells, moderately expressing αvβ3-integrins, and negative control 293T cells (Figure 8b,c). In HeLa cells transfection efficacy of ligand-modified RGD1, RGD2 and RGD3-complexes was compared with that of RGD0/DNA polyplexes that can be explained by decreased αvβ3-expression level in HeLa cells. According to Figure 7b, chloroquine addition to transfection media led to an increase in transgene activity mediated by RGD1-complexes only. In chloroquine-containing media, efficiency of RGD1/DNA polyplexes was significantly higher (8–16-fold) than efficiency of control RGD0/DNA complexes. It could be suggested that in HeLa an endosomolitic activity of the RGD1 carrier was not high enough to provide an efficient gene delivery. However, the increased level of transgene expression after chloroquine treatment along with data on cellular uptake of RGD1-polyplexes by HeLa cells (Figure 7) reflects an efficient receptor targeting by the iRGD ligand and reveals dependence between the surface level of receptor and transfection efficiency. Chloroquine addition during transfection experiments with RGD2/DNA and RGD3/DNA complexes in HeLa cells did not lead to a significant increase in transgene expression. Moreover, the polyplexes’ efficacy was compared with that of control RGD0/DNA complexes even in the presence of the endosomolitic agent. Lower transfection efficiency of ligand-modified complexes in HeLa cells correlated with the lower level of αvβ3-expression in the cells. The results are consistent with those of cellular uptake study when the cell penetration efficiency of polyplexes decreased along with a decreasein the αvβ3-expression level (Figure 7). Similar findings confirming correlation of receptor surface level and transfection efficiency have been shown previously [36,37]. Additionally, the transfection efficacy of ligand-modified DNA-complexes in 293T cells was virtually the same as that of unmodified polyplexes both in the absence and presence of the endosomolitic agent (Figure 8c). This finding is consistent with the observation that 293T cells poorly express αvβ3 integrins. The decrease in the efficacy of some complexes in the presence of chloroquine can be an evidence of their toxic effect on cells. A similar effect has been described previously [35]. Thus, the results obtained confirm a high specificity of RGDs carriers in targeted DNA delivery and are consistent with data from other studies [34].

Further, targeted gene delivery by RGD carriers was assessed by the inhibitory effect of a free cyclic RGD ligand in transfection studies. We conducted a competitive transfection of PANC-1 cells with the most efficient RGD1/DNA complexes at an 8/1 charge ratio in the presence of a free cyclo(RGDfK) peptide (Figure 8e). We found that c(RGDfK) peptide pre-treatment of the cells resulted in a significant decrease in the RGD1/DNA polyplexes’ efficacy. The demonstrated inhibitory effect of the free cyclo(RGDfK) peptide indicates that the iRGD moiety is involved in internalization of the complexes through αvβ3 integrins and it functions as expected. 

### 2.8. Proliferative Activity of Cancer Cells after Suicidal Gene Delivery

**Figure 9:**
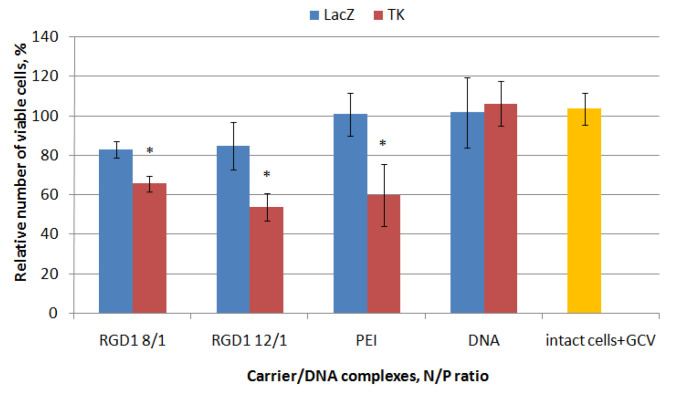
Thymidine kinase-driven suicide gene therapy in vitro: the PANC-1 cell line was transfected with RGD1/DNA polyplexes at charge ratios of 8/1 and 12/1, pPTK-1 or pCMVlacZ plasmids alone and PEI/DNA. The transfection experiments were performed in the presence of ganciclovir (GCV). Values are the mean ±SD of the mean of triplicate experiments. * *p* < 0.05 compared to carrier/pCMVlacZ complexes.

The therapeutic effect of herpes simplex virus (HSV-1) thymidine kinase gene delivery mediated by the RGD1-carrier followed by ganciclovir treatment was studied in PANC-1 cells representing a model of pancreatic ductal adenocarcinoma [38]. RGD1/pPTK-1 polyplexes were studied at 8/1 and 12/1 charge ratios. Similar polyplexes formed with pCMV-lacZ plasmid were taken as a negative control. Ganciclovir treatment did not affect the viability of non-transfected cells as well as “naked” DNA delivery (Figure 9). We found that transfection of PANC-1 cells with RGD1/pCMV-lacZ polyplexes led to insignificant decrease in their proliferation in comparison with intact cells that can be explained by a toxic effect of the carrier itself. In other hand, transfection with pPTK-1 plasmid resulted in a 34–46% decrease inthe cell viability because of response to ganciclovir treatment (Figure 9). Transfection with PEI/pPTK-1polyplexes was taken as a positive control and resulted in 40% decrease in the viability of PANC-1 cells. Significant differences in the cell viability between RGD1-polyplexes bearing pCMV-lacZ or pPTK-1 plasmid confirm effect of suicide gene therapy in vitro.

### 2.9. In Vitro GFP and VEGFA Gene Expression Silencing by Carrier/siRNA Complexes

The effects of anti-GFP siRNA delivery mediated by the RGD1-carrier on GFP protein production were investigated in GFP + MDA-MB-231 cells representing a model of late-stage breast cancer [39]. According to the literature data, the integrin αvβ3 is moderately expressed in MDA-MB-231 which makes it possible to use this cell line for targeted siRNA delivery by theiRGD-modified carrier [40,41]. We supposed that the siRNA-polyplexes would enter the cells via thecaveolae-mediated internalization pathway due to their increased sizes. Previously, it was shown that the endocytosis of integrin αvβ3 may occur by clathrin dependent and clathrin independent endocytic mechanisms, including a caveolin 1 dependent route [13]. The internalization by caveolae-mediated endocytosis is known not to be associated with a decrease in pH and leads to the avoidance of lysosomal degradation, so chloroquine was not used during siRNA transfection experiments to test endosomolitic properties [42].

**Figure 10:**
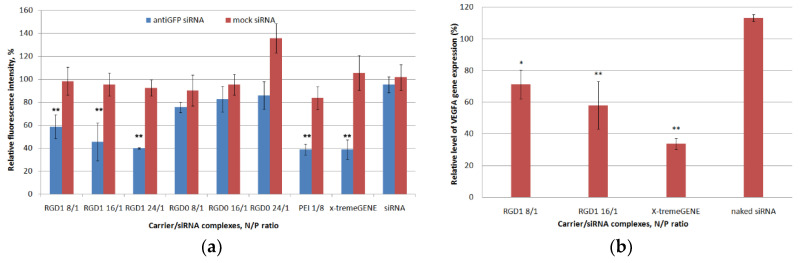
siRNA transfection efficacy evaluation: (**a**) reduction of GFP production after treatment of MDA-MB-231 cells with carrier/siRNA complexes. ** *p* < 0.01 when compared with cells treated by naked anti-GFP siRNA. The data are shown as the mean ± S.E.M. (**b**) reduction of VEGFA gene expression after treatment of E.A.Hy926 cells with carrier/siRNA complexes. The level of VEGFA gene expression after delivery of mock siRNA was taken as 100%. * *p* < 0.05, ** *p* < 0.01 when compared with cells treated by naked mock siRNA. The data are shown as the mean ± S.E.M.

Both anti-GFP and mock siRNA were used to demonstrate specificity of GFP gene expression silencing by RNAi. The silencing efficiency was measured at N/P ratios of 8/1, 16/1 and 24/1 (RGD1 and RGD0 carriers) and 8/1 (PEI), respectively. RGD2 and RGD3 carriers were not tested as vehicles for siRNA delivery because of their sub-optimal DNA transfection and uptake capacity (Figure 7 and Figure 8). An anti-GFP and mock siRNA only treatment was used as a negative control. As shown in Figure 10a, the negative control did not show down-regulation of GFP gene expression and protein production. The cell treatment by RGD1/anti-GFP-siRNA complexes at N/P ratios of 8/1, 16/1 and 24/1 resulted in a decrease inGFP production to 58%, 45% and 40%, respectively, whereas RGD0/anti-GFP-siRNA polyplexes suppressed GFP only up to 76%, 83% and 86%, respectively (Figure 10a). Thus, it can be suggested that polyplexes with RGD ligand content are predominantly internalized in MDA-MB-231 cells via iRGD ligand-mediated endocytosis, which resulted in more efficient GFP silencing, whereas ligand-free RGD0/siRNA polyplexes are likely to internalize via nonspecific absorptive endocytosis. It is important to notice that GFP gene suppression by RGD1/anti-GFP-siRNA complexes at N/P ratios of 24/1 was at the same level as control PEI/anti-GFP-siRNA polyplexes and x-tremeGENE/anti-GFP-siRNA lipoplexes. However, the complexes bearing control mock siRNA did not induce any silencing compared to anti-GFP siRNA-bearing complexes. This result allows the suggestion that the suppression of GFP gene expression was achieved by means of the specific RNAi effect but not from the carriers’ toxicity.

RNAi-mediated down-regulation of VEGFA gene expression was achieved in the EA.Hy926 cell line (hybridoma of primary HUVEC and A549 cells) which reproduces the main morphological, phenotypical and functional features of the endothelium [43]. VEGFA is a key regulator of angiogenesis and silencing of its expression is a promising approach to anti-angiogenic therapy of cancer and some other diseases related to pathological angiogenesis e.g., choroidal neovascularization and endometriosis [4,44,45]. The most efficient RGD1/siRNA polyplexes formed at 8/1 and 16/1 charge ratios were tested for their ability to down-regulate gene expression. Mock siRNA-bearing polyplexes were used as a control to ensure involvement of RNAi ingene expression silencing and their values were taken as 100%. According to the results in Figure 10b, VEGFA gene expression was decreased by RGD1-mediated anti-VEGFA siRNA delivery by 28.74% and 41.9%, respectively. Previously, we have shown that similar silencing of VEGFA gene expression resulted in significant anti-angiogenic effects in endothelial cell modeland in rat model of endometriosis [44,46]. Thus, RGD1-polyplexes can be suggested as siRNA-carriers in anti-angiogenic therapeutic RNAi.

## 3. Materials and Methods

### 3.1. Cell Lines

Human cervical carcinoma (HeLa), human kidney (293T), human pancreatic (PANC-1) cell lines (from Institute of Cytology RAS, Saint-Petersburg, Russia), GFP-expressing human breast cancer cell line MDA-MB 231 (kind gift of Prof. Jessica Rosenholm, Abo Academy University, Turku, Finland) and endothelial cells EA.hy926 (kind gift of Dr. Cora-Jean C. Edgell, University of North Carolina, USA) were maintained under mycoplasma-free conditions as described previously [43,47].

### 3.2. Peptide Synthesis

R_9_H_4_CRGDRGPDC (RGD1), R_9_H_4_ (RGD0) and cyclo(RGDfK) (free ligand) peptides were synthesized using a solid phase Boc-chemistry by NPF Verta, LLC (Saint-Petersburg, Russia), analysed by MALDI TOF (Figure S3; Table S1), supplied as a dry powder, and stored desiccated at −20 °C. Quantities of 1–2 mg of RGD0 or cyclo(RGDfK) were dissolved in dH_2_O at 2 mg/mL and stored as small aliquots at −20 °C. RGD1 was dissolved in 1–2 mg quantities in 0.5 mM Hepes (pH = 7.5) at 0.1 mg/mL and allowed to stand overnight at room temperature for cyclization through two cysteines. SDS-PAGE was performed to confirm the correct cyclization of the RGD1 peptide (Figure S4). Then it was concentrated to 2 mg/mL by means of rotor evaporator (Labconco), frozen in small aliquots and stored at −70 °C. The purity of the peptides determined by high-performance liquid chromatography was not less than 95% (Figure S5). Amount of free thiol groups in cyclic RGD1 was estimated by Ellman’s assay at 0 h and 24 h after cyclization (Figure S6) [37].

### 3.3. Plasmids and siRNAs

The pCMV-lacZ plasmid encoding β-galactosidase (kind gift of B. Sholte, Erasmus University Rotterdam, The Netherlands), pEXPR-IBA5-eGFP plasmid encoding GFP (IBA GmbH, Göttingen, Germany) and pPTK1 plasmid encoding herpes simplex virus (HSV-1) thymidine kinase gene (kind gift of S. Orlov, Institute of Experimental Medicine, Saint-Petersburg, Russia) were purified using a Qiagen Plasmid Giga kit under endotoxin free conditions (Qiagen, Dorking, UK) as described previously [37]. The pDNA was diluted in water to 0.5–1 mg/mL and stored at −20 °C.

The sense strand of anti-GFP siRNA 5′-CAA GCU GAC CCU GAA GUU Ctt-3′ targets GFP mRNA [47]. The sense strand of anti-VEGFA siRNA 5′-GCG GAU CAA ACC UCA CCA Att-3′ targets human VEGFA transcript [43]. A non-silencing siRNA 5′-UUC UCC GAA CGU GUC ACG U- 3′ served as a mock siRNA. siRNAs were purchased from Syntol JSC, Moscow, Russia.

### 3.4. Preparation of DNA- and RNA-Complexes

DNA/peptide complexes were formed at different charge ratios (N/P—peptide NH3+/DNA PO4- ratio). The molar N/P charge ratio of RGD1 and RGD0 peptides to negatively charged PO4− groups in DNA was calculated considering that 1 mg of cationic RGD1 and RGD0 peptides corresponds to 3.1 nmol and 4.56 nmol of positive charges, respectively. The required amount of pDNA was diluted in 0.05 mg/mL in Hepes-buffered mannitol (HBM) (5% *w*/*v* mannitol, 5 mM Hepes, pH 7.5). The peptides at 2 mg/mL in HBM were added to pDNA solution and vortexed for 30 sec, and then the complexes were left for 30 min. The carriers RGD2 and RGD3 were obtained by mixing of RGD1 and RGD0 peptides (50 mol% and 50 mol%; 10 mol% and 90 mol%, respectively) before the formation of DNA/peptide complexes.

siRNA/peptide complexes were prepared at various N/P ratios in the range 0.5–24. The appropriate volume of the peptide carrier (2 mg/mL) was added to the siRNA solution (0.05 mg/mL) in HBM and vortexed. Then, the polyplexes were allowed to stand at room temperature for 30 min.

Polyethyleneimine (branched PEI 25 kDa; Sigma) was used as a 0.9 mg/mL (pH 7.5) aqueous stock solution stored at 4 °C. The ratio of PEI to DNA and RNA was 8/1. x-tremeGENE transfection reagent (Roche) was used for siRNA delivery according to the manufacturer recommendation at a weight ratio of 1/5.

### 3.5. DNA- and RNA-Binding Assays

Peptide-binding to DNA was monitored using the ethidium bromide (EtBr) fluorescence quenching method as described previously [48]. Peptide binding to siRNA was studied with the SYBR-Green fluorescence quenching method [43]. Fluorescence measurements were performed in a Wallac 1420D scanning multilabel counter (PerkinElmer Wallac Oy, Turku, Finland). Displacement was calculated as (F −Ff)/(Fb −Ff), where Ff and Fb are the fluorescence intensities of EtBr or SYBR-Green in the absence and presence of pDNA or siRNA, respectively.

### 3.6. DNase I and RNase A Protection Assays

In total, 20μLof the peptide/DNA complexes were prepared as described above and incubated with 0.5 units of DNase I (Ambion) for 30 min at 37 °C. Then DNase I inactivation reagent (Ambion, Waltham, MA, USA) was added for 2 min to inactivate DNase I. In total, 8 μL of the peptide/siRNA complexes were prepared as described above and incubated with 100 ng of RNase A (BioChemica AppliChem, Darmstadt, Germany) for 30 min at 37 °C. Then 1% SDS was added for 5 min at 98 °C to inactivate RNase A. To release DNA and RNA, the complexes were treated overnight with trypsin (0.1%) at 37 °C. Thereafter, DNA was analyzed by 0.8% agarose gel electrophoresis and siRNA was analyzed by 15% polyacrylamide (PAA) gel electrophoresis where PAA gel was stained by AgNO_3_ [49]. The integrity of DNA or RNA was compared with native DNA and DNase I treated DNA or native siRNA and RNase A treated RNA, respectively.

### 3.7. Measurement of Size and Z-Potential of Peptide/DNA and Peptide/RNA Complexes

The peptide/DNA and peptide/RNA complexes were prepared as described above in quantities of 5 μg of DNA or RNA per sample and at an N/P ratio of 8. The size of the complexes was determined using dynamic light scattering, and the zeta potential was determined by the micro electrophoresis. Three independent measurements were performed on a zetasizer NANO ZS (Malvern Instruments, Malvern, UK).

### 3.8. Relaxation of DNA- and RNA-Complexes by Dextran-Sulfate

The peptide/DNA and peptide/siRNA complexes were prepared as described above in the NA-binding section. Then dextran-sulfate (DS) (Sigma, USA) was added to the complexes at three-fold charge excess relative to the peptide. After 24 h of incubation, EtBr or SYBR-Green fluorescence was measured by means of aWallac 1420D scanning multilabel counter at appropriate wavelengths and dye displacement was calculated.

### 3.9. Expression of αvβ3 Integrins in Cell Lines

αvβ3 integrin expression in PANC-1, HeLa and 293T cells was determined using a BD FACS-Canto II cytofluorimeter (Becton-Dickinson Biosciences, Franklin Lakes, NJ, USA) by means of FITC-labeled mouse anti-human CD51/CD61 antibodies (BD Pharmingen, San Jose, CA, USA). 10,000 living cells were taken into account.

### 3.10. Gene Transfer

Transfection experiments were performed in triplicate. PANC-1, HeLa or 293T cells were seeded at a density of 5.0 × 10^4^ cells/well in 48-well plates a day before the experiment. Cell culture medium was replaced with medium without fetal bovine serum (FBS) before transfection. DNA complexes were added and incubated with cells for 4 h. Then the transfection medium was removed, fresh FBS-supplemented medium was added and the cells were incubated for the next 48 h. The final amount of DNA was 2 mkg in each well. The β-galactosidase activity in cell extracts was measured with methyl-umbelliferyl-β-D-galactopyranoside (MUG) as described previously [37]. The lacZ gene expression was normalized by the total protein concentration of the cell extracts, measured with Bradford reagent (Helicon, Moscow, Russia). For the endosomolytic activity study DNA complexes were incubated with cells for 4 h in the presence of 100 µM chloroquine (Sigma, Ronkonkoma, NY, USA). In control experiments complexes were incubated with cells in the absence of chloroquine. A 10-fold excess of cyclo(RGDfK) peptide was added to PANC-1 cells 15 min before complex treatment for the competition study. DNA/peptide complexes were added to medium containing free cyclo(RGDfK) peptide and incubated for 4 h. In control experiments, complexes were incubated with cells without the cyclo(RGDfK) peptide. GFP expression was determined by flow cytometry with a BD FACS-Canto II cytofluorimeter at 48 h after the transfection of PANC-1 cells. Transfection efficacy is reported as a percentage of GFP-positive cells.

### 3.11. Cellular Uptake of Peptide/DNA Complexes

PANC-1, HeLa or 293T cells were seeded at a density of 6 × 10^4^ cells/well in 48-well plates. Peptide/DNA complexes were prepared with YOYO-1 iodide (1 molecule of the dye per 50 base pairs). Transfection was performed as described above. After 2 h of the complexes’ treatment, the cells were washed twice in 1× PBS (pH 7.2) and once with 1M NaCl (in 1× PBS). Then the cells were detached, resuspended and incubated with propidium iodide solution (50 µg/mL in 1× PBS) for 15 min in the dark to exclude dead cells. Subsequently, the cells were processed by flow cytometry with a BD FACS-Canto II cytofluorimeter. The results were presented as RFU/cell. A total of 10,000 living cells were taken into account.

### 3.12. Cytotoxicity Assay

The cytotoxicity of DNA/peptide complexes was evaluated in PANC-1, HeLa, 293T and MDA-MB-231 cells in 96-well plates using Alamar blue assay (BioSources International, San Diego, CA, USA) for cell viability after 16 h of incubation as described previously [37]. The amount of DNA was 0.7 mkg per well and siRNA concentration was 200 nM, similarly to transfection experiments. The fluorescence was recorded on a Wallac 1420D scanning multilabel counter with an excitation wavelength of 544 nm and an emission wavelength 590 nm. The relative fluorescence intensity was counted according to (F − Ff)/(Fb − Ff) × 100%, where Fb and Ff are the fluorescence intensities in untreated control and without cells, respectively.

### 3.13. Analysis of Proliferative Activity of PANC-1 Cells after Suicidal Gene Therapy

The efficacy of suicide gene therapy after delivery of pPTK-1 plasmid and pCMV-lacZ (as a control) was accessed in PANC-1 cells in the presence of ganciclovir as previously reported [50]. Briefly, PANC-1 cells were placed into 96-well cultural plates at a rate of 1.5 × 10^4^ cells per well in the standard culture medium, grown for 24 h, and washed with the FBS-free medium. The fresh FBS-free medium and the DNA/carrier complex suspension (0.7 μg of pPTK1 or pCMV-lacZ plasmid per well) were applied. The plates were incubated for 2 h and washed with FBS-free medium. Then the standard culture medium was added to each well and the plate was incubated for 24 h. The medium was substituted with the standard culture medium with 50 μg/mLof ganciclovir, and the plate was incubated for 96 h before cytotoxicity measurement by Alamar blue assay as described above.

### 3.14. siRNA Transfer to MDA-MB-231 Cells and GFP Fluorescence Detection

The siRNA transfection experiments were done in triplicate. A total of 5 × 10^4^ cells wereseeded in 48-well plates and incubated overnight. The transfections were performed in FBS-free medium. The complexes bearing anti-GFP siRNA or mock siRNA were incubated with cells for 4 h (200 nM per well). After 48 h incubation in FBS-supplemented medium, cells were washed by 1× PBS (pH 7.2) and permeabilized with the reporter cell lysis buffer. GFP fluorescence was measured at an excitation wavelength of 485 nm and an emission wavelength of 535 nm by means of a Wallac 1420D scanning multilabel counter. The GFP fluorescence level was normalized relative to the total protein concentration in each sample measured using Bradford reagent.

### 3.15. siRNA Transfer to EA.hy926 Cells and Quantitative RT-PCR

Transfection experiments in EA.hy926 cells were performed in triplicate as previously described [46]. After incubation in a fully supplemented cell culture medium for 48 h, cells were taken for RNA extraction. Total RNA extraction and quantitative real-time PCR analysis were performed as previously described [43]. The following primers were used: VEGFA forward primer 5′-GAGCTAAAAATCTTGACCCACATTG-3′, reverse primer 5′-CAGTATTCAACAATCACCATCAGAG-3′; and endogenous reference gene β-actin expression was measured using forward 5′-TGCCGACAGGATGCAGAAG-3′, reverse primer 5′-GCCGATCCACACGGAGTACT-3′. The samples were measured three times and a final result was inferred by averaging the data. The values are presented as means ± S.E.M of the means obtained from three independent experiments.

### 3.16. Statistical Analysis

Statistical analysis was performed using the Mann–Whitney U-test and Student’s *t*-test by means of GraphPad Prism 6 software package (GraphPad Prism Inc., San Diego, CA, USA). Differences with *p* < 0.05 and *p* < 0.01 were considered statistically significant.

## 4. Conclusions

The present work describes iRGD ligand-conjugated peptide carriers for tumor targeted DNA and siRNA delivery. The complexes of DNA and αvβ3 integrin-targeted carriers demonstrated highly specific uptake by the cancer cells with a correlation between an efficiency of the polyplexes’ uptake and the amount of αvβ3 integrins on the cells’ surface. The RGD1 carrier efficiently delivered DNA and siRNA into αvβ3 integrin-expressing cancer cells with a relatively low cytotoxicity level. A competition transfection assay with a free cyclic RGD ligand proved a ligand-mediated gene delivery by the RGD1 carrier. These results, taken together, allow us to conclude that the developed peptide carrier RGD1 modified with a tumor targeting iRGD ligand can be considered as promising candidate for in vivo study as a DNA and siRNA delivery system for cancer gene therapy.

## Figures and Tables

**Table 1:** Design of the carriers.

Name	Composition (mol%)
RGD0	RRRRRRRRRHHHH (100 mol%)
RGD1	RRRRRRRRRHHHH-CRGDRGPDC (100 mol%) |__________|
RGD2	RRRRRRRRRHHHH (50 mol%) RRRRRRRRRHHHH-CRGDRGPDC (50 mol%) |__________|
RGD3	RRRRRRRRRHHHH (90 mol%) RRRRRRRRRHHHH-CRGDRGPDC (10 mol%) |__________|

## Supplementary Materials

The following are available online at https://www.mdpi.com/1424-8247/13/10/300/s1, Figure S1: Detection of αvβ3 integrins on the surface of 293T, HeLa, PANC-1 cell lines by flow cytometry analysis, Figure S2: Cytotoxicity evaluation of free carriers in 293T, HeLa, PANC-1, and MDA-MB-231 cells by the Alamar blue assay, Figure S3: Typical MALDI TOF mass spectra of RGD0 and RGD1 peptides, Figure S4: SDS-PAGE results for RGD0 and RGD1 peptides, Figure S5: Typical HPLC results for RGD0 and RGD1 peptides, Figure S6: Free thiol groups measurement by Ellman’s assay after 24 h of RGD1 peptide cyclization, Table S1: Experimental and theoretical molecular masses of RGD0 and RGD1 peptides.

## References

[1] Ginn S.L., Amaya A.K., Alexander I.E., Edelstein M., Abedi M.R. (2018). Gene therapy clinical trials worldwide to 2017: An update. J. Gene Med..

[2] Belitsky G.A., Kirsanov K.I., Lesovaya E.A., Yakubovskaya M.G. (2019). Prevention of therapy-related malignances in cancer survivors. Oncotarget.

[3] Pahle J., Walther W. (2016). Vectors and strategies for nonviral cancer gene therapy. Expert Opin. Biol. Ther..

[4] Li T., Kang G., Wang T., Huang H.E. (2018). Tumor angiogenesis and anti - angiogenic gene therapy for cancer. Oncol. Lett..

[5] Dunbar C.E., High K.A., Joung J.K., Kohn D.B., Ozawa K., Sadelain M. (2018). Gene therapy comes of age. Science.

[6] Egorova A., Kiselev A. (2016). Peptide modules for overcoming barriers of nucleic acids transport to cells. Curr. Top. Med. Chem..

[7] Bansal A. (2018). Non-Viral Vectors for Gene Delivery. Nanosci. Nanotechnol. -Asia.

[8] Wang D., Tai P.W.L., Gao G. (2019). Adeno-associated virus vector as a platform for gene therapy delivery. Nat. Rev. Drug Discov..

[9] Taylor R.E., Zahid M. (2020). Cell Penetrating Peptides, Novel Vectors for Gene Therapy. Pharmaceutics.

[10] Futaki S. (2005). Membrane-permeable arginine-rich peptides and the translocation mechanisms. Adv. Drug Deliv. Rev..

[11] Midoux P., Monsigny M. (1999). Efficient gene transfer by histidylated polylysine/pDNA complexes. Bioconjug. Chem..

[12] Amreddy N., Babu A., Muralidharan R., Panneerselvam J., Srivastava A., Ahmed R., Mehta M., Munshi A., Ramesh R. (2018). Recent Advances in Nanoparticle-Based Cancer Drug and Gene Delivery. Advances in Cancer Research.

[13] Caswell P.T., Vadrevu S., Norman J.C. (2009). Integrins: Masters and slaves of endocytic transport. Nat. Rev. Mol. Cell Biol..

[14] Huang R., Rofstad E.K. (2018). Integrins as therapeutic targets in the organ-specific metastasis of human malignant melanoma. J. Exp. Clin. Cancer Res..

[15] Ruoslahti E. (2017). Tumor penetrating peptides for improved drug delivery. Adv. Drug Deliv. Rev..

[16] Temming K., Schiffelers R.M., Molema G., Kok R.J. (2005). RGD-based strategies for selective delivery of therapeutics and imaging agents to the tumour vasculature. Drug Resist. Updates.

[17] Fu S., Xu X., Ma Y., Zhang S., Zhang S. (2019). RGD peptide-based non-viral gene delivery vectors targeting integrin α v β 3 for cancer therapy. J. Drug Target.

[18] Aoki Y., Hosaka S., Kawa S., Kiyosawa K. (2001). Potential tumor-targeting peptide vector of histidylated oligolysine conjugated to a tumor-homing RGD motif. Cancer Gene Ther..

[19] Teesalu T., Sugahara K.N., Kotamraju V.R., Ruoslahti E. (2009). C-end rule peptides mediate neuropilin-1-dependent cell, vascular, and tissue penetration. Proc. Natl. Acad. Sci. USA.

[20] Lo J.H., Hao L., Muzumdar M.D., Raghavan S., Kwon E.J., Pulver E.M., Hsu F., Aguirre A.J., Wolpin B.M., Hahn W.C. (2018). iRGD-guided Tumor-penetrating Nanocomplexes for Therapeutic siRNA Delivery to Pancreatic Cancer. Mol. Cancer Ther..

[21] Bjorge J.D., Pang A., Fujita D.J. (2017). Delivery of gene targeting siRNAs to breast cancer cells using a multifunctional peptide complex that promotes both targeted delivery and endosomal release. PLoS ONE.

[22] Xin Y., Huang M., Guo W.W., Huang Q., zhen Zhang L., Jiang G. (2017). Nano-based delivery of RNAi in cancer therapy. Mol. Cancer.

[23] Kim K., Hwang H.S., Shim M.S., Cho Y.Y., Lee J.Y., Lee H.S., Kang H.C. (2019). Controlling complexation/decomplexation and sizes of polymer-based electrostatic pDNA polyplexes is one of the key factors in effective transfection. Colloids Surf. B Biointerfaces.

[24] Nisakar D., Vij M., Pandey T., Natarajan P., Sharma R., Mishra S., Ganguli M. (2019). Deciphering the Role of Chondroitin Sulfate in Increasing the Transfection Efficiency of Amphipathic Peptide-Based Nanocomplexes. ACS Biomater. Sci. Eng..

[25] Egorova A., Shubina A., Sokolov D., Selkov S., Baranov V., Kiselev A. (2016). CXCR4-targeted modular peptide carriers for efficient anti-VEGF siRNA delivery. Int. J. Pharm..

[26] Rejman J., Oberle V., Zuhorn I.S., Hoekstra D. (2004). Size-dependent internalization of particles via the pathways of clathrin- and caveolae-mediated endocytosis. Biochem. J..

[27] Xu L., Anchordoquy T. (2011). Drug Delivery Trends in Clinical Trials and Translational Medicine: Challenges and Opportunities in the Delivery of Nucleic Acid-Based Therapeutics. J. Pharm. Sci..

[28] Taherian A., Li X., Liu Y., Haas T.A. (2011). Differences in integrin expression and signaling within human breast cancer cells. BMC Cancer.

[29] Kim H.A., Nam K., Kim S.W. (2014). Tumor targeting RGD conjugated bio-reducible polymer for VEGF siRNA expressing plasmid delivery. Biomaterials.

[30] Shen H., Huang X., Min J., Le S., Wang Q., Wang X., Dogan A.A., Liu X., Zhang P., Xiao J. (2019). Nanoparticle delivery systems for DNA/RNA and their potential applications in nanomedicine. Curr. Top. Med. Chem..

[31] Swenson S., Ramu S., Markland F. (2007). Anti-Angiogenesis and RGD-Containing Snake Venom Disintegrins. Curr. Pharm. Des..

[32] Pouton C.W., Seymour L.W. (2001). Key issues in non-viral gene delivery1PII of original article: S0169-409X(98)00048-9. The article was originally published in Advanced Drug Delivery Reviews 34 (1998) 3–19.1. Adv. Drug Deliv. Rev..

[33] Bono N., Ponti F., Mantovani D., Candiani G. (2020). Non-Viral in vitro Gene Delivery: It is Now Time to Set the Bar!. Pharmaceutics.

[34] Wonder E., Simón-Gracia L., Scodeller P., Majzoub R.N., Kotamraju V.R., Ewert K.K., Teesalu T., Safinya C.R. (2018). Competition of charge-mediated and specific binding by peptide-tagged cationic liposome–DNA nanoparticles in vitro and in vivo. Biomaterials.

[35] Guryanov I.A., Vlasov G.P., Lesina E.A., Kiselev A.V., Baranov V.S., Avdeeva E.V., Vorob’ev V.I. (2005). Cationic oligopeptides modified with lipophilic fragments: Use for DNA delivery to cells. Russ. J. Bioorg. Chem..

[36] Kircheis R., Kichler A., Wallner G., Kursa M., Ogris M., Felzmann T., Buchberger M., Wagner E. (1997). Coupling of cell-binding ligands to polyethylenimine for targeted gene delivery. Gene Ther..

[37] Egorova A., Bogacheva M., Shubina A., Baranov V., Kiselev A. (2014). Development of a receptor-targeted gene delivery system using CXCR4 ligand-conjugated cross-linking peptides. J. Gene Med..

[38] Rouanet M., Lebrin M., Gross F., Bournet B., Cordelier P., Buscail L. (2017). Gene therapy for pancreatic cancer: Specificity, issues and hopes. Int. J. Mol. Sci..

[39] Welsh J. (2013). Animal Models for Studying Prevention and Treatment of Breast Cancer. Animal Models for the Study of Human Disease.

[40] Stojanović N., Dekanić A., Paradžik M., Majhen D., Ferenčak K., Ruščić J., Bardak I., Supina C., Tomicic M.T., Osmak M. (2018). Differential Effects of Integrin α v Knockdown and Cilengitide on Sensitization of Triple-Negative Breast Cancer and Melanoma Cells to Microtubule Poisons. Mol. Pharmacol..

[41] Zuo H. (2019). iRGD: A Promising Peptide for Cancer Imaging and a Potential Therapeutic Agent for Various Cancers. J. Oncol..

[42] Kagaya H., Oba M., Miura Y., Koyama H., Ishii T., Shimada T., Takato T., Kataoka K., Miyata T. (2012). Impact of polyplex micelles installed with cyclic RGD peptide as ligand on gene delivery to vascular lesions. Gene Ther..

[43] Egorova A.A., Maretina M.A., Kiselev A.V. (2019). VEGFA Gene Silencing in CXCR4-Expressing Cells via siRNA Delivery by Means of Targeted Peptide Carrier. Methods Mol. Biol..

[44] Egorova A., Petrosyan M., Maretina M., Balashova N., Polyanskih L., Baranov V., Kiselev A. (2018). Anti-angiogenic treatment of endometriosis via anti-VEGFA siRNA delivery by means of peptide-based carrier in a rat subcutaneous model. Gene Ther..

[45] Ryoo N.K., Lee J., Lee H., Hong H.K., Kim H., Lee J.B., Woo S.J., Park K.H., Kim H. (2017). Therapeutic effects of a novel siRNA-based anti-VEGF (siVEGF) nanoball for the treatment of choroidal neovascularization. Nanoscale.

[46] Egorova A.A., Shtykalova S.V., Maretina M.A., Sokolov D.I., Selkov S.A., Baranov V.S., Kiselev A.V. (2019). Synergistic Anti-Angiogenic Effects Using Peptide-Based Combinatorial Delivery of siRNAs Targeting VEGFA, VEGFR1, and Endoglin Genes. Pharmaceutics.

[47] Slita A., Egorova A., Casals E., Kiselev A., Rosenholm J.M. (2018). Characterization of modified mesoporous silica nanoparticles as vectors for siRNA delivery. Asian J. Pharm. Sci..

[48] Kiselev A.V., Il’ina P.L., Egorova A.A., Baranov A.N., Guryanov I.A., Bayanova N.V., Tarasenko I.I., Lesina E.A., Vlasov G.P., Baranov V.S. (2007). Lysine dendrimers as vectors for delivery of genetic constructs to eukaryotic cells. Russ. J. Genet..

[49] Beidler J.L., Hilliard P.R., Rill R.L. (1982). Ultrasensitive staining of nucleic acids with silver. Anal. Biochem..

[50] Egorova A.A., Shtykalova S.V., Maretina M.A., Selyutin A.V., Shved N.Y., Krylova N.V., Ilina A.V., Pyankov I.A., Freund S.A., Selkov S.A. (2020). Cys-flanked cationic peptides for cell delivery of the herpes simplex virus thymidine kinase gene for suicide gene therapy of uterine leiomyoma. Mol. Biol..

