# Supplementary material for: Characterization of iRGD-Ligand Modified Arginine-Histidine-Rich Peptides for Nucleic Acid Therapeutics Delivery to αvβ3 Integrin-Expressing Cancer Cells
Source: Pharmaceuticals (Basel). 2020 Oct 10;13(10):300. doi: 10.3390/ph13100300 (PMC7601072; doi:10.3390/ph13100300)
Supplement: Supplementary file 1 [file pharmaceuticals-13-00300-s001.pdf]

Supporting Information for the Article

Characterization of iRGD-ligand modified arginine-histidine-rich peptides for nucleic acid therapeutics delivery to αvβ3 integrin-expressing cancer cells

Anna Egorova [1,3], Alexander Selutin [2], Marianna Maretina [1,3], Sergei Selkov [2], Vladislav Baranov [1] and Anton Kiselev [1,*]

[1] Department of Genomic Medicine, D.O. Ott Research Institute of Obstetrics, Gynecology and Reproductology, Mendeleevskaya line 3, 199034 Saint-Petersburg, Russia; egorova_anna@yahoo.com (A.E.); marianna0204@gmail.com (M.M.); baranov@vb2475.spb.edu (V.B.)

[2] Department of Immunology and Intercellular Interactions, D.O. Ott Research Institute of Obstetrics, Gynecology and Reproductology, Mendeleevskaya line 3, 199034 Saint-Petersburg, Russia; a_selutin@yahoo.com (A.S.); selkovsa@mail.ru (S.S.)

[3] Institute of Chemistry, Saint Petersburg State University, Universitetskii pr. 26, 198504 Peterhoff, Russia;

* Correspondence: ankiselev@yahoo.co.uk (A.V); Tel.: +7-812-328-9809

Contents

Figure S1. The flow cytometric detection of $\alpha v\beta 3$ integrins on the surface of 293T, HeLa, and PANC-1 cell lines....3

Figure S2. Cytotoxicity evaluation of free carriers in 293T, HeLa, PANC-1, and MDA-MB-231 cells....4

Figure S3. MALDI TOF mass spectra of the peptides....5

Table S1. Experimental and theoretical molecular masses of the peptides synthesized....6

Figure S4. SDS-PAGE results for the peptides....7

Figure S5. HPLC results for the peptides....8

Figure S6. Free thiol groups measurement by Ellman's assay after 24 h of RGD1 peptide cyclization....9

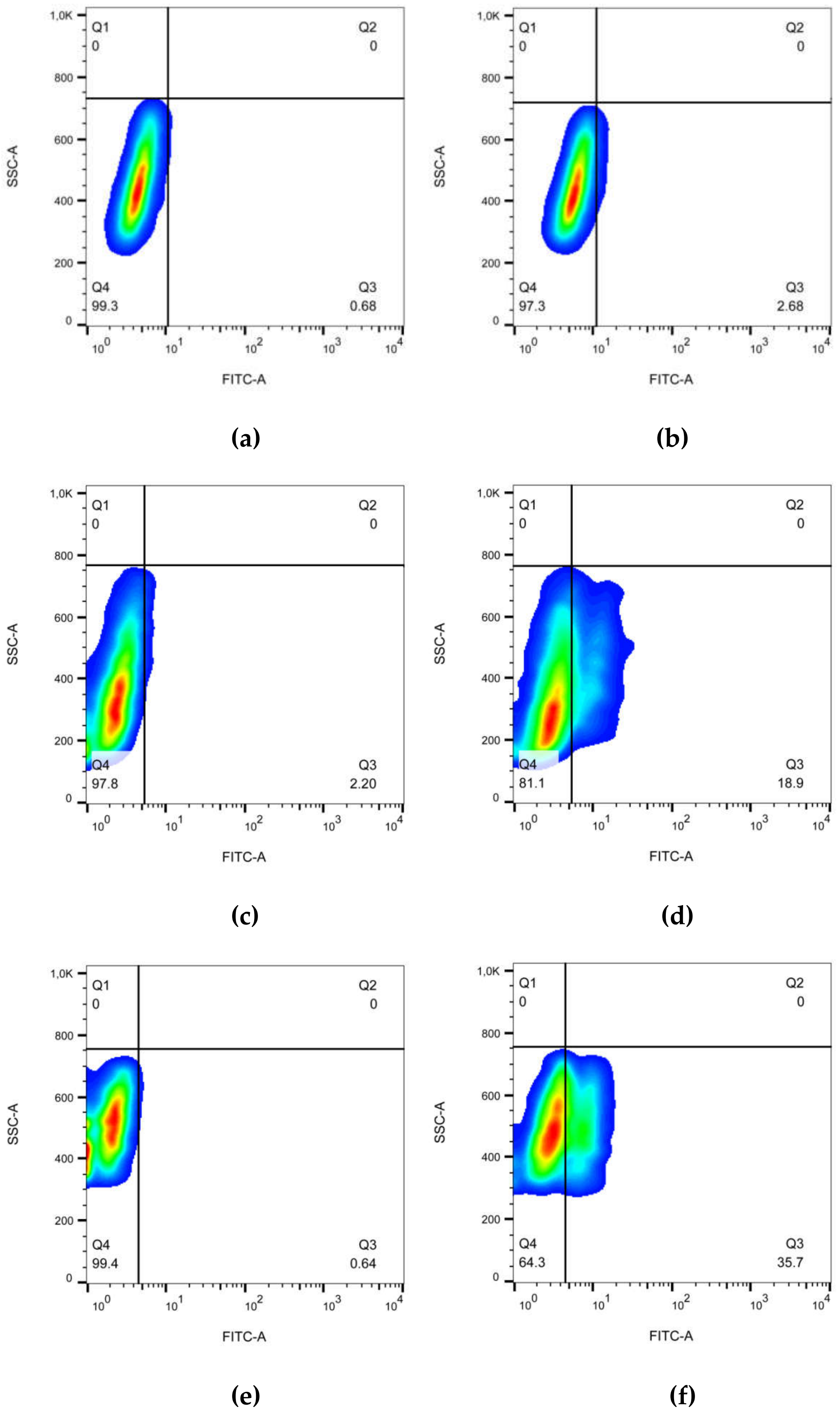

Figure S1. Detection of αvβ3 integrins on the surface of 293T **(a,b)**, HeLa **(c,d)**, PANC-1 **(e,f)** cell lines by flow cytometry analysis. **(a,c,e)** - unstained cells; **(b,d,f)** - CD51/CD61 antibody staining. The FACS data were processed by FlowJo software (FlowJo, LLC).

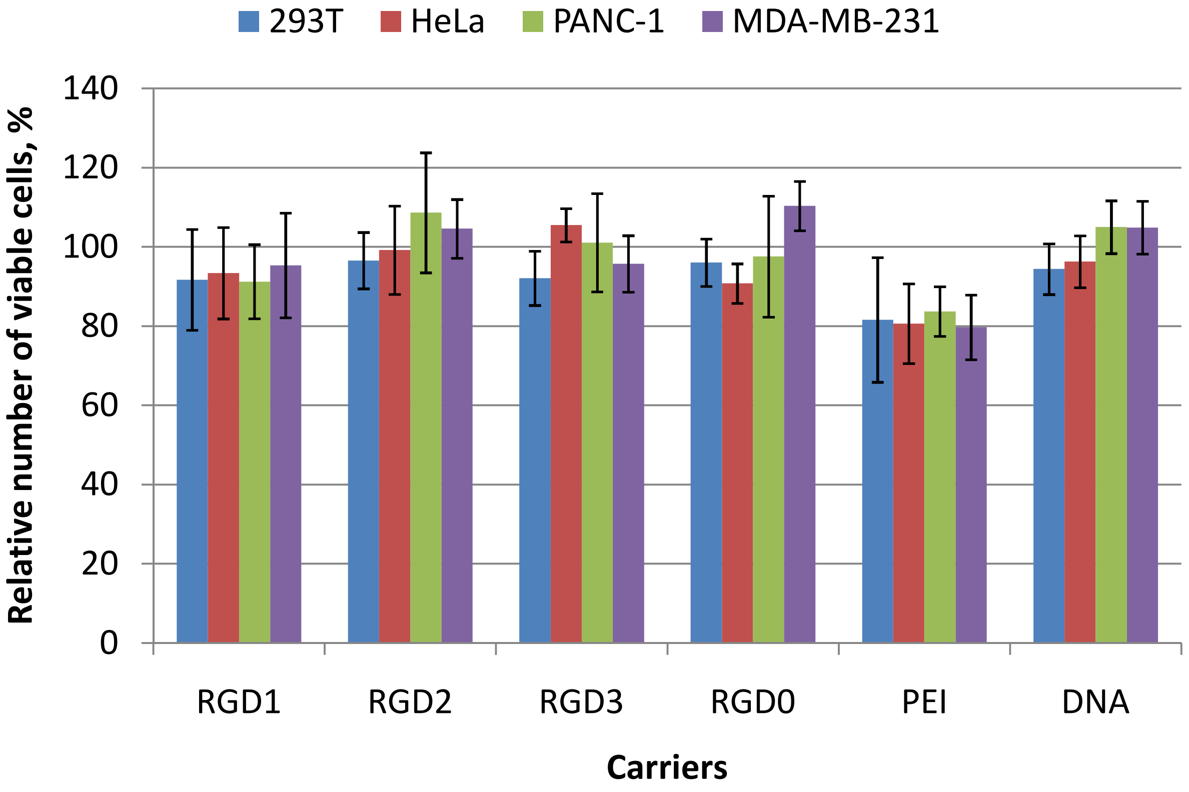

Figure S2. Cytotoxicity evaluation of free carriers in 293T, HeLa, PANC-1 , and MDA-MB-231 cells by the Alamar blue assay. Values are the mean ± SD of the mean of triplicates. Amounts of carriers tested correspond to those applied for formation of NA/carriers complexes at N/P ratio 12/1.

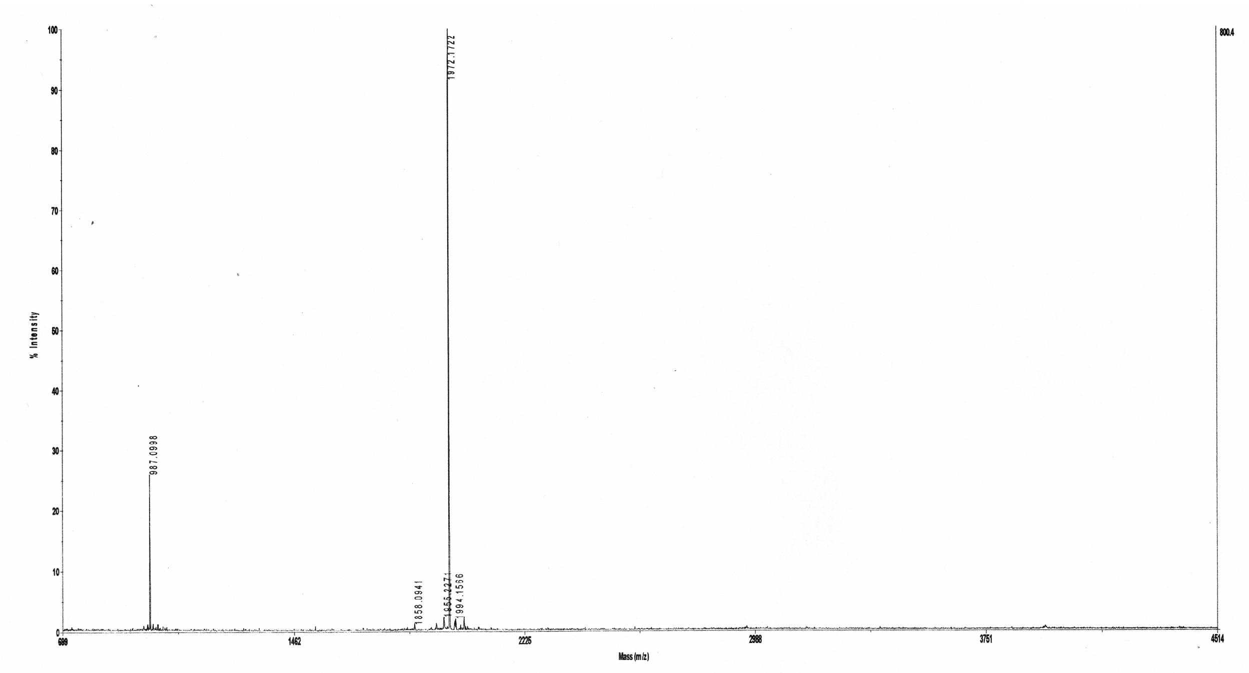

(a)

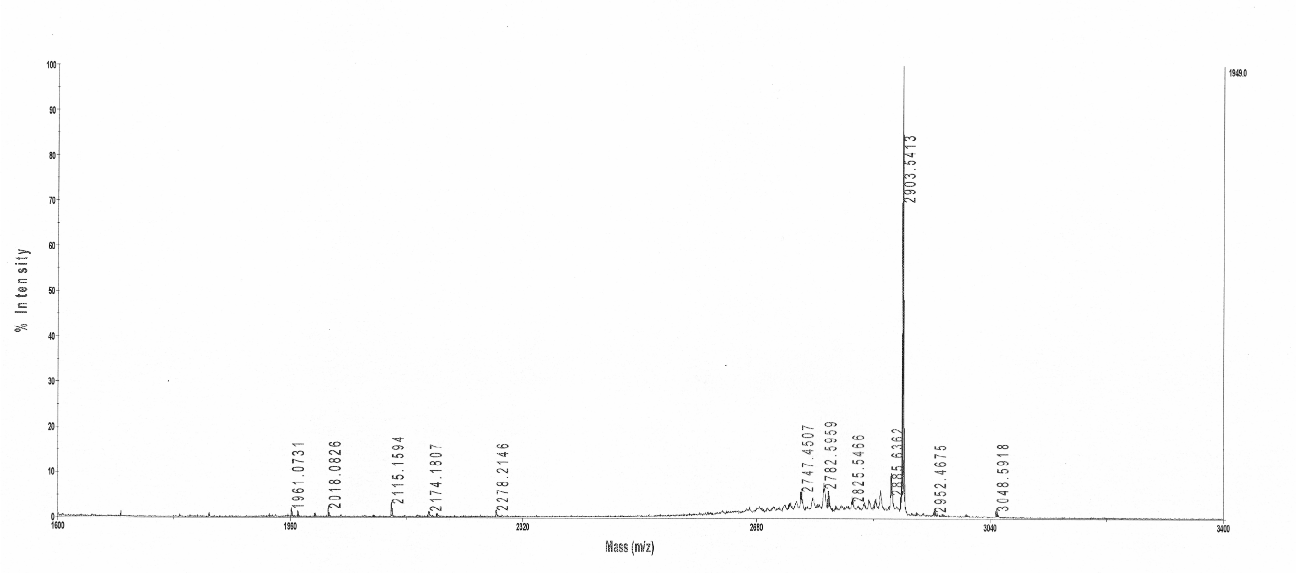

(b)

Figure S3. Typical MALDI TOF mass spectra of RGD0 **(a)** and RGD1 **(b)** peptides.

Table S1. Experimental and theoretical molecular masses of RGD0 and RGD1 peptides.

Peptide analyzed	Calculated value of molecular mass, g/mole	Experimental value of molecular mass, g/mole
RGD0	1972.28	1972.17
RGD1	2904.33	2903.54

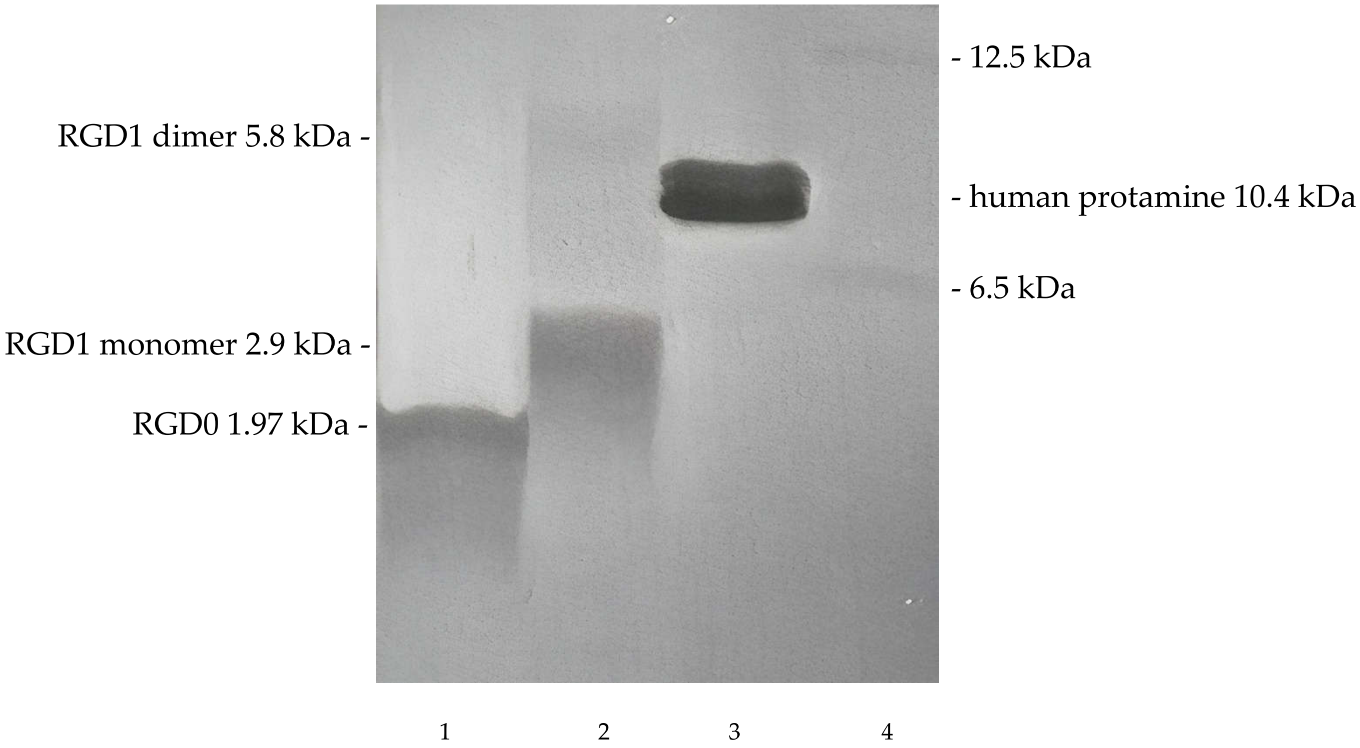

Figure S4. SDS-PAGE results for RGD0 **(1)** and RGD1 **(2)** peptides; **(3)** - human protamine 1 protein; **(4)** - protein test mixture 6 for SDS PAGE (SERVA).

Note 1: RGD1 dimer cannot be fully denatured by SDS and possesses changed electrophoretic mobility.

Note 2: Tricine-sodium dodecyl sulphate-polyacrylamide gel electrophoresis 4%/16.5% (tricine-SDS-PAGE) was performed to separate low-molecular-weight peptides. Human protamine 1 protein (10.4 kDa) and protein test mixture 6 for SDS PAGE (SERVA) were used as molecular weight controls. Coomassie Brilliant Blue was used for the gel staining.

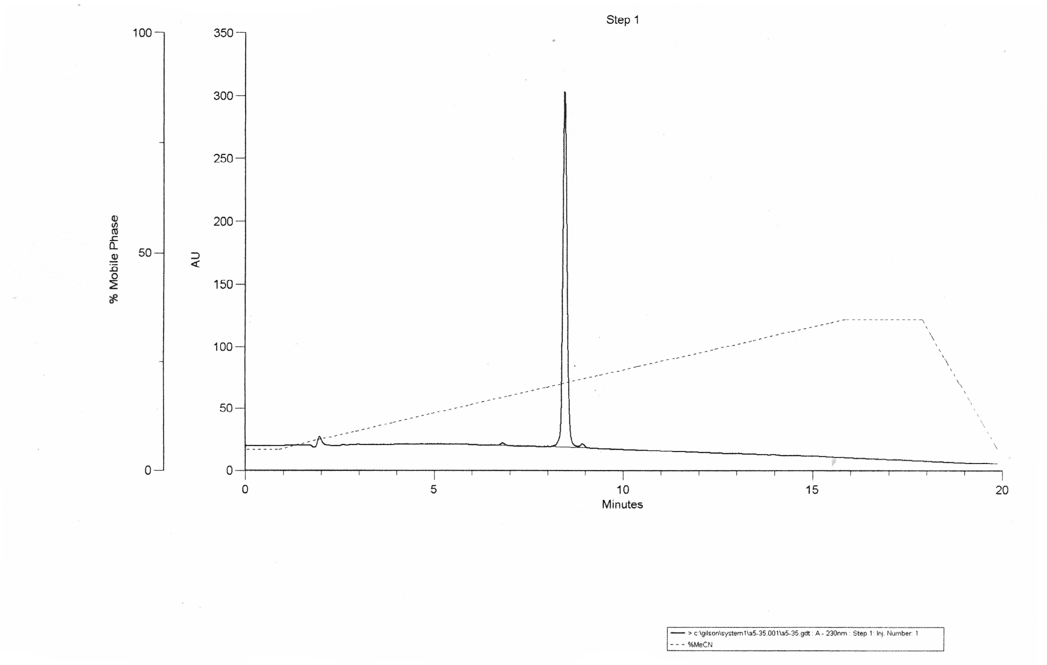

	R. Time	Area	Area %					
1	6.80	28880	0.751					
2	8.45	3766444	97.946					
3	8.92	50099	1.303					

(a)

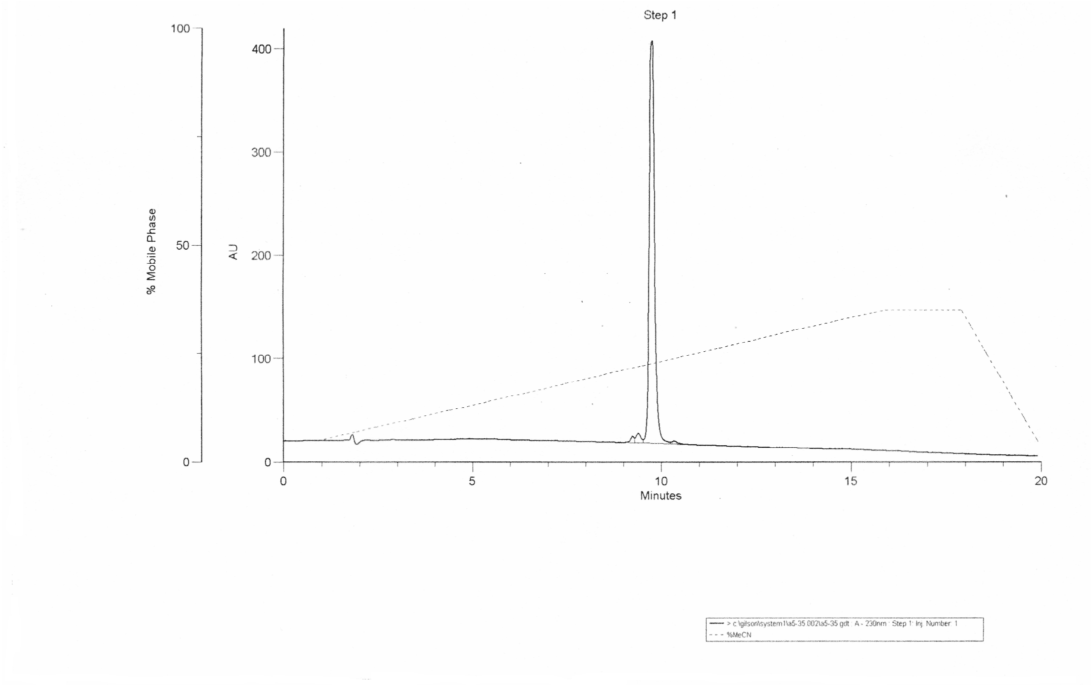

	R. Time	Area	Area %					
1	9.24	72863	1.077					
2	9.40	142790	2.111					
3	9.77	6485236	95.867					
4	10.34	63908	0.945					

(b)

Figure S5. Typical HPLC results for RGD0 **(a)** and RGD1 **(b)** peptides.

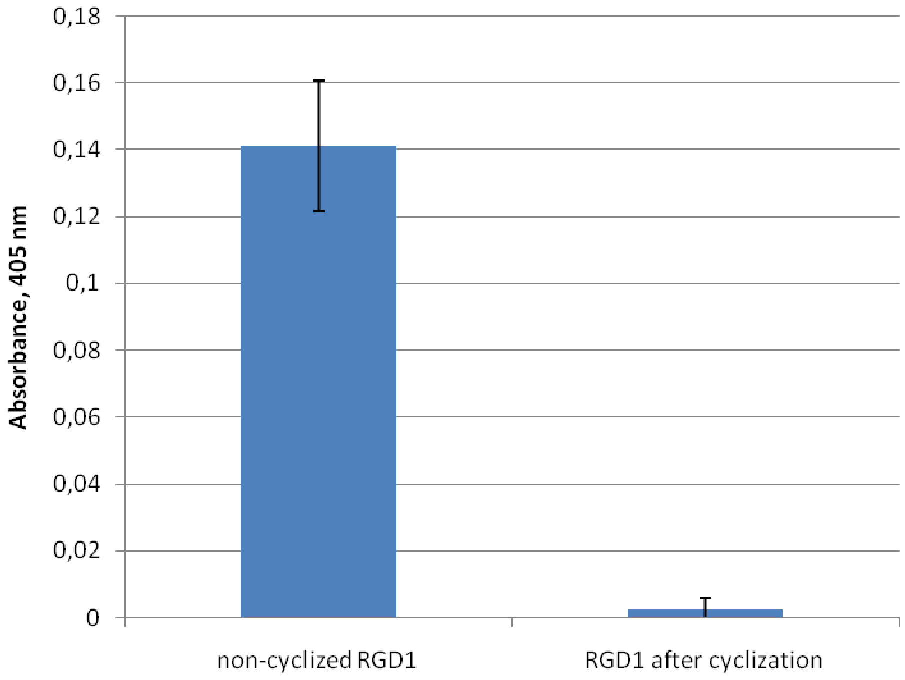

Figure S6. Free thiol groups measurement by Ellman's assay after 24 h of RGD1 peptide cyclization.
